# Physical Education Attitude of Adolescent Students in the Philippines: Importance of Curriculum and Teacher Sex and Behaviors

**DOI:** 10.3389/fpsyg.2021.658599

**Published:** 2021-03-29

**Authors:** Angelita B. Cruz, Minsung Kim, Hyun-Duck Kim

**Affiliations:** ^1^Department of Physical Education, Keimyung University, Daegu, South Korea; ^2^School of Sport for All, Kyungwoon University, Gumi, South Korea; ^3^Department of Sport Marketing, Keimyung University, Daegu, South Korea

**Keywords:** Filipino students, student-teacher interaction, physical education, MODE model, physical education attitude scale

## Abstract

The present study examined the attitudes of Filipino middle school students toward physical education (PE) and the associations between PE attitude and various personal and external correlates of PE. In total, 659 middle school students, aged between 12 and 19 years (M = 14.55; SD = 1.14), participated in the study. The Physical Education Attitude Scale (PEAS) was used to measure affective, cognitive, and motivational/behavioral attitudes of adolescent students toward PE. Results showed that middle school students had moderate general attitudes toward PE. Female students had more favorable attitudes toward PE when their teacher was male than female. When the teacher was female, male students were more satisfied with the PE curriculum than female students. When the teacher was male, female students were more comfortable with the PE curriculum than male students. Finally, students' PE attitude did not decrease as they got older, regardless of student sex. The findings provide a different perspective for the field and underscore the importance of not only the PE curriculum but also the student–teacher relationship. To prevent the decline in students' positive attitude and encourage positive behaviors toward PE and activities, teachers should be very considerate about their interactions with students of the same sex; school administrators (e.g., principal and PE department head), meanwhile, should focus at providing PE teachers with special training courses to enhance both their teaching and communication capabilities.

## Introduction

People's different views about a target object may affect how they respond to it; these views are often categorized as either positive or negative. In school, for instance, students can have a positive or negative attitude toward certain subjects, and this can occur owing to various reasons. Some students may have a positive attitude toward physical education (PE), for example, because they like the activities they play in the class (Luke and Sinclair, 1991), while others can have negative attitudes toward PE because they dislike the way the teacher manages the class or the activity itself (Luke and Sinclair, 1991). These developed attitudes toward PE, in turn, can either be detrimental or favorable to students' current and future participation in PE, mainly because attitudes people have toward a particular object, person, or thing are said to influence behaviors (Ajzen, 1985, p. 11–39; Fazio and Olson, 2014, p. 155–171).

Based on the motivation and opportunity (MODE) model (Fazio and Olson, 2014, p. 155–171), attitude is defined as individuals' association of the “attitude object” and the overall evaluation of this attitude object that is stored in their memory. The evaluation of the attitude object depends on individuals' perceived views, feelings, and prior experiences, or even various combinations of these cognitive, emotional, and behavioral sources of information, all of which are elicited from individuals' memory. This evaluation may vary in valence (i.e., either positive or negative), and the strength of the association between the attitude object and its evaluation, especially a strong one, tends to be automatically activated from individuals' memory upon encountering the attitude object; this process eventually determines individuals' judgment and/or behavior in an immediate situation. To counter the direct influence of a strongly activated attitude, individuals should have the motivation and opportunity to deliberately process the attitude object.

Accordingly, students who have experienced enjoyable physical activities (PA) in the PE class are likely to have learned meaningful lessons and to have felt other pleasant events related to PE; these experiences and the resulting memories may yield a strong positive attitude toward PA and PE, and thereby, strong participation. The reverse may be true for students who have not found PA and PE enjoyable in the past. Thus, memory affects students' behavior in an ongoing way. Based on this understanding—which is backed up by the MODE model—we can deem as important the examination of students' attitudes toward PE and which aspects of PE classes they prefer or dislike, in order to revise those activities to improve their effectiveness at building positive memories and engaging students.

Students' attitude toward PE (or PE attitudes) have been shown to be associated with various personal and external factors. Regarding personal factors, some researchers have found female students to have more positive attitudes toward PE than male students (Zeng et al., 2011; Pereira et al., 2020), while others found opposite results (Koca and Demirhan, 2004; Koca et al., 2005; Mercier et al., 2017; Orlić et al., 2017), and other studies have found PE attitudes to be similar between sexes (Subramaniam and Silverman, 2007; Scrabis-Fletcher et al., 2016; Marttinen et al., 2018). Meanwhile, studies that examined the association between grade level and PE attitude showed a decline in students' positive attitudes toward PE as grade increased (Subramaniam and Silverman, 2007; Hu et al., 2014; Silverman, 2017; Pereira et al., 2020). Furthermore, extracurricular sports participation has also been shown to influence students' PE attitudes, with students involved in sports showing more positive attitudes than those not involved (Koca and Demirhan, 2004; Orlić et al., 2017; Pereira et al., 2020).

Regarding external factors, PE curriculum, class atmosphere, and teacher behaviors have all been shown to affect students' PE attitude (Luke and Sinclair, 1991; Rikard and Banville, 2007; Subramaniam and Silverman, 2007). Exemplifying, curriculum content that promotes a fun and enjoyable atmosphere was shown to impact the development of positive attitudes among students (Rikard and Banville, 2007; Subramaniam and Silverman, 2007; Dismore and Bailey, 2011); however, curriculum content can also influence the formation of negative attitudes toward PE, particularly when it comprises long runs or repetitive and boring PE-related activities (Luke and Sinclair, 1991; Rikard and Banville, 2007). Furthermore, teachers' behaviors, especially their evaluation and decision-making methods, are found to highly impact students' negative attitudes toward PE (Luke and Sinclair, 1991).

While a plethora of studies regarding students' attitude toward PE and its correlates are available—as shown above—most previous studies focused on the affective and cognitive views of attitudes and on students with Western backgrounds. In particular, researchers who previously examined PE attitude of students utilized the Students' Attitudes toward Physical Education Questionnaire (SAtPE; Subramaniam and Silverman, 2000) anchored on the affective and cognitive components of attitudes. While the instrument is widely accepted, others (Orlić et al., 2017) presented its limitations and developed the PE Attitude Scale (PEAS) as an alternative assessment tool that examines a broader spectrum of aspects of PE experiences within the affective, cognitive, as well as motivational/behavioral indicators of attitude. Furthermore, albeit studies found that teachers' behaviors greatly affected students' negative attitude toward PE, the influence of teacher sex was not considered (Luke and Sinclair, 1991); this variable can also be an influential factor on students' PE attitude. Correlatively, a study found that male students perceived that their female teachers provided more time/feedback/attention to them than to their female peers (Nicaise et al., 2007). Thus, further investigation is warranted regarding students' attitude toward PE with consideration to motivational/behavioral indicators of attitude and teacher sex as dependent and independent variables, respectively.

In the Philippines, a country in Southeast Asia, empirical findings about students' PE attitude are almost nonexistent. Concomitantly, Filipino students have recently been found to have the second highest prevalence of insufficient PA among school-aged adolescents worldwide (World Health Organization, 2019). Thus, extending the scientific setting to this country seems important because identifying Filipino students' PE attitude and its associated factors can provide valuable insights; specifically, Filipino stakeholders on the development of enhanced policies and curricula that can promote PE as a positive academic subject can make good use of such knowledge for their endeavors. In doing so, Filipino students may incur in better outcomes regarding their participation in sports and other PAs, thereby decreasing their risks of future health problems.

For that reason, this study aimed to examine Filipino middle school students' attitude toward PE and the associations between their PE attitudes and various personal and external correlates of PE. Particularly, we analyzed the correlates of PE and their individual relative contributions to PE attitude by sex. We also examined students' PE attitude differences by sex, grade level, PA outside school, and PE teacher sex. Accordingly, we put forward the following hypotheses:

Hypothesis 1: Filipino middle school students will report a moderately positive attitude toward PE;
Hypothesis 2: Various known correlates of PE will have significant relationships with PE attitude;
Hypothesis 3: PE attitude levels will differ by student sex;
Hypothesis 4: Students with pre-existing PA outside school will show more positive attitudes toward PE than those with no such experience;
Hypothesis 5: Students' attitude toward PE will decrease as grade level increases; and, based on existing literature,
Hypothesis 6: PE teacher sex will influence students' attitude toward PE positively or negatively.

## Materials and Methods

### Participants

In total, 659 middle school students, aged between 12 and 19 years (M = 14.55; SD = 1.14), partook in this study. The sample is composed of 37.5% grade 8, 29% grade 9, and 22% grade 10 students. There were 43.6% male and 56.4% female students. Moreover, 48.5% and 51.5% of the sample students had either male or female teacher, respectively. Through a non-probabilistic convenience sampling method, participants were recruited from six classes in grade 8, 7 classes in grade 9, and 5 classes in grade 10. All participants studied in a public national high school in an urban city in the Philippines.

### Instrument

The Physical Education Attitude Scale (PEAS) was used to measure adolescent students' attitude toward PE (Orlić et al., 2017). It comprises 43 items that measure a wide array of experiences regarding PE and are rated on a five-point Likert scale (1, *strongly disagree*; 5, *strongly agree*). It is divided into the following four subscales: *satisfaction* (12 items; e.g., “I like to attend PE classes”), relating to general emotions about PE experiences; *comfort* (12 items; e.g., “I feel uncomfortable in PE classes”), relating to specific feelings about PE; *activity* (11 items; e.g., “I like to show what I know in PE classes”), relating to motivational processes when participating in PE classes; and *teacher* (8 items; e.g., “PE teacher encourages me in class”), relating to perceptions about the PE teacher. The psychometric properties and construct and external validity of the PEAS were supported (Orlić et al., 2017). In this study, its overall Cronbach's α was of 0.92; that for its subscales was 0.83, 0.76, 0.81, and 0.73, in the order of satisfaction, comfort, activity, and teacher, respectively. The principal component correlations in this study ranged from 0.350 to 0.555.

### Data Collection Procedure

Prior to data collection, we obtained approval from the school principal and PE teachers; specifically, the researchers conducted a session with all PE teachers to explain the objectives and procedures of the study. The main researcher read and explained each item in the questionnaire to PE teachers and provided clarifications to any questions about any aspect of the study or its questionnaire. Then, 15–20 min during the beginning of the scheduled PE class, the PE teachers informed their students about the research and explained its objectives. Students were briefed about participation being purely voluntary and that anonymity would be ensured. After, PE teachers distributed the questionnaires to their respective students and performed the same instruction protocol as previously directed by the researcher. In addition to their own informed consent, students were also requested, if considered minors, to obtain written approval from their parents/guardians prior to answering the questionnaire. To encourage honest and truthful responses, students were notified that their answers would be strictly confidential and the results would only be used for academic purposes. Students were given 1 week to return the questionnaires. Submitted survey forms were sorted and checked for incomplete responses. The procedures of this study followed both the ethical principles put forward by the Declaration of Helsinki regarding human participants and those of the national psychological association of the Philippines where the research was conducted.

### Data Analysis

Data were encoded via Microsoft Excel and were cross-checked for errors in encoding or missing values. Data were discarded if the following occurred: relevant information in the questionnaire was missing or incomplete; statements were not properly answered; and/or showed straightforward responses. The data were then exported to SPSS for further data screening tests (e.g., tests for normality and outliers). From the original 664 samples, data from 659 students were qualified for statistical analysis.

To examine the influence of student sex, grade level, and the interactions between student and teacher sex on PE attitudes, we conducted multivariate analysis of variance (MANOVA). Upon the appearance of significant differences between factor levels, we conducted *post hoc* analysis; to compare the main and interaction effects, we conducted pairwise *t*-tests with Bonferroni adjustments. Last, to predict students' general PE attitude based on correlates of PE, as well as the relative contribution of each correlate by student sex, we conducted hierarchical linear regression analysis. All significance levels were set to a *p* < 0.05.

## Results

### Descriptive Analysis

Overall, (see Table 1) middle school students reported moderately positive attitudes toward PE (3.82 ± 0.48). Male and female students had similar average scores for PE attitude. Likewise, grade 8 and grade 9 students expressed moderately positive attitudes toward PE; however, grade 10 students reported very strong positive attitudes toward PE (4.06 ± 0.50).

**Table 1:** Overall PE attitude and dimensions of PE scores based on student sex, teacher, sex, grade level, and physical activity outside school.

		**Satisfaction**		**Comfort**		**Activity**		**Teacher**		**PE Attitude**	
		**Mean**	**SD**	**Mean**	**SD**	**Mean**	**SD**	**Mean**	**SD**	**Mean**	**SD**
**Overall**		3.93	0.57	3.70	0.56	3.92	0.55	3.70	0.59	3.82	0.48
**Student sex**											
Male students		3.89	0.62	3.70	0.60	3.94	0.58	3.71	0.63	3.82	0.52
Female students		3.96	0.53	3.70	0.53	3.91	0.52	3.69	0.56	3.83	0.44
**Grade level**											
8		3.82	0.55	3.61	0.55	3.84	0.52	3.56	0.62	3.72	0.44
9		3.89	0.53	3.67	0.53	3.89	0.53	3.66	0.56	3.79	0.45
10		4.19	0.59	3.91	0.59	4.12	0.58	4.00	0.50	4.06	0.50
**PA outside school**											
With		3.94	0.54	3.71	0.54	3.92	0.54	3.69	0.58	3.82	0.46
Without		3.89	0.58	3.64	0.57	3.92	0.55	3.68	0.61	3.79	0.47
**Student sex**	**Teacher sex**										
Male students	Male	3.89	0.60	3.72	0.61	3.99	0.56	3.83	0.57	3.86	0.52
	Female	3.89	0.64	3.67	0.58	3.88	0.61	3.56	0.67	3.77	0.51
Female students	Male	4.08	0.54	3.84	0.52	3.99	0.50	3.80	0.51	3.94	0.44
	Female	3.87	0.51	3.60	0.53	3.85	0.53	3.61	0.58	3.74	0.43

*PE, physical education; PA, physical activity*.

Moreover, 75.5% of the students were involved in PA outside school. However, students had similar favorable attitudes toward PE regardless of PA outside school status. Furthermore, students' PE attitudes for male (48.5%) and female (51.5%) PE teachers were favorable and almost alike (average range: 3.74–3.94 ± 0.43–0.52).

### Effects of Personal and External Factors on PE Attitude

Results of MANOVA showed a statistically significant univariate effect of grade level on PE attitude [Pillai's Trace = 0.047, *F*_(5,581)_ = 2.78, *p* < 0.002; partial η^2^ = 0.023]. However, PA outside school, student sex, and teacher sex did not influence PE attitude.

A discriminant analysis for grade level showed differences for general PE attitude [*F*_(2,585)_ = 6.40, *p* < 0.01; partial η^2^ = 0.021] and in the dimensions of satisfaction [*F*_(2,585)_ = 6.97, *p* < 0.01; partial η^2^ = 0.023], activity [*F*_(2,585)_ = 3.82, *p* < 0.05; partial η^2^ = 0.013], and teacher [*F*_(2,585)_ = 5.67, *p* < 0.01; partial η^2^ = 0.019]. Multiple comparisons between grade levels showed that the mean scores for general PE attitude (4.13 vs. 3.72/3.79), satisfaction (4.25 vs. 3.78/3.90), activity (4.21 vs. 3.86/3.89), and teacher (4.07 vs. 3.56/3.67) were statistically significant between grade 10 and grades 8 and 9. The mean score for the comfort dimension was non-significantly higher in grade 10 than in the other two grades, whereas the mean scores for all variables between grades 9 and 8 were not statistically different.

The interaction effects between the independent variables and PE attitude did not show any statistical significance, except for the interaction between student and teacher sex [Pillai's Trace = 0.020, *F*_(5,581)_ = 2.37, *p* < 0.05; partial η^2^ = 0.020]. A discriminant analysis for the interaction between student and teacher sex showed differences in general PE attitude [*F*_(1,585)_ = 6.04, *p* < 0.01; partial η^2^ = 0.010] and in the dimensions of satisfaction [*F*_(1,585)_ = 7.11, *p* < 0.01; partial η^2^ = 0.012] and comfort [*F*_(1,585)_ = 7.84, *p* < 0.01; partial η^2^ = 0.013]. Multiple pairwise comparisons showed that, when the teacher was male, the scores for general PE attitude (3.90 vs. 3.74) and the comfort dimension (3.84 vs. 3.60) were substantially higher in female than in male students. When the teacher was female, the satisfaction score of male students was statistically higher than that of female students (4.11 vs. 3.85).

### Regression Analyses for the Predictors of PE Attitude by Student Sex

For the hierarchical regression analyses, Block 1 included age (representing grade level), teacher sex, PA outside school; Block 2 also included the PE dimensions (see Table 2).

**Table 2:** Hierarchical multiple regression predicting PE attitude.

**Variable**	**Cumulative**		**Simultaneous**	
	***R*****^**2**^ change**	**F change**	**β**	***p***
**Male students**				
Block 1	0.034	F_(3,256)_ = 3.02^*^		
Teacher sex			−0.001	0.222
Age			0.000	0.296
PA-POS			0.000	0.233
Block 2	0.966	F_(4,252)_ = 1,493,540.89^**^		
Teacher			0.237	<0.001^**^
Activity			0.294	<0.001^**^
Comfort			0.324	<0.001^**^
Satisfaction			0.328	<0.001^**^
**Female students**				
Block 1	0.047	F_(3,338)_ = 5.58^**^		
Teacher sex			0.000	0.883
Age			−0.001	0.215
PA-POS			0.001	0.025^*^
Block 2	0.953	F_(4,334)_ = 1,360,480.20^**^		
Teacher			0.235	<0.001^**^
Activity			0.303	<0.001^**^
Comfort			0.336	<0.001^**^
Satisfaction			0.338	<0.001^**^

Teacher sex: 1 = male, 2 = female; PA-POS, Physical Activity Participation Outside School: 1 = with physical activity, 2 = without physical activity; β = standardized beta coefficient; p = significance value of all predictors in the final model;

* p < 0.05,

** *p < 0.001*.

Concerning male students, the regression model generally explained all the variability in PE attitude [*R*^2^ = 1.00, *F*_(7,252)_ = 883,676.365, *p* < 0.001]. Teacher sex, age, and PA outside school predicted ~3.4% of the variance in PE attitude, but were not found to be significant predictors of PE attitude in the final model. After controlling for these factors, the variables introduced in Block 2 were shown to predict ~96.6% of the variance in PE attitude; specifically, all PE dimensions significantly predicted PE attitude, in that higher scores for satisfaction (β = 0.33), comfort (β = 0.32), activity (β = 0.29), and teacher (β = 0.24) were associated with a more positive attitude toward PE.

Concerning female students, the regression model generally explained all the variability in PE attitude [*R*^2^ = 1.00, *F*_(7,334)_ = 815,938.716, *p* < 0.001]. Teacher sex, age, and PA outside school predicted ~4.7% of the variance in PE attitude, but only PA outside school was a significant predictor in the final model; specifically, students with no PA outside school had a more positive attitude toward PE. After controlling for the factors in Block 1, the variables introduced in Block 2 were shown to predict ~95.3% of the variance in PE attitude; particularly, all PE dimensions significantly predicted PE attitude, in that higher scores for satisfaction (β = 0.34), comfort (β = 0.31), activity (β = 0.29), and teacher (β = 0.24) were associated with a more positive attitude toward PE.

After analyzing potential multicollinearity for the study variables, we found that issues regarding this topic were not a concern; the VIF scores were lower than the recommended limit of 10 (Myers, 1990).

## Discussion

This study aimed to investigate Filipino middle school students' attitude toward PE and the associations between PE attitude and various personal and external correlates of PE. In particular, we analyzed the correlates of PE and their individual relative contribution to PE attitude by student sex. We also examined the influences of sex, grade level, PA outside school, and teacher sex on students' attitude toward PE.

The mean scores of PE attitude and its dimensions ranged from 3.70 to 3.93 in our sample, indicating that students had moderate positive attitudes toward PE. This result confirms our hypothesis 1, suggesting that Filipino students generally have pleasant experiences and feelings toward PE and perceive the subject as valuable. These findings corroborate Orlić et al. (2017)'s research, who examined PE attitude of Serbian students using PEAS; they found that the mean scores for PE attitude (3.81) and the dimensions of satisfaction (3.82), activity (3.64), and teacher (3.55) were moderately positively high. Nonetheless, in their study, the comfort dimension showed a higher score (mean score of 4.14). In a review of meaningful experiences in PE and youth sports (Beni et al., 2017), social interaction, fun, and challenge were considered as important contributors of meaningful experiences in PE. Thus, the difference between our results and those of Orlić et al. (2017) regarding the comfort dimension (i.e., moderate vs. high) may be due to Serbian students being able to experience more pleasant social interactions and to have more fun and/or more challenging activities in their PE class; this could have made them feel more relaxed and less anxious, leading to the higher positive attitude in the comfort dimension than that of our sample.

In another study that used PEAS to measure PE attitude (Orlić et al., 2018), the result showed that PE attitude of students was very high (4.21). This difference in results may be attributed to the characteristics of the students who likely experienced and enjoyed better PE class and thereby reported higher positive attitude in all dimensions of PE yielding a higher overall evaluation compared to our findings. However, specific scores of each PE dimension were not reported; thus, it is difficult to distinguish the source(s) that contributed to students' positive evaluation toward PE. Nevertheless, PE attitudes of students in our study were still positively similar to previous findings. While literatures about PE attitude are available, studies that examine PE attitude using PEAS are still limited. Hence, more studies are suggested, utilizing this instrument tool in assessing PE attitude to further understand general and specific information in the development of positive or negative attitude toward PE class.

### Influence of Student and Teacher Sex, Grade Level, and PA Outside School on PE Attitudes

The findings based on grade level revealed that PE attitude of grade 10 students was significantly more positive than those of grade 8 and 9 students. Furthermore, students' positive attitudes toward PE did not decline as grade level increased, but rather showed an increasing trend. These findings refuted hypothesis 5 and contrasted previous works (Subramaniam and Silverman, 2007; Hu et al., 2014; Silverman, 2017; Pereira et al., 2020). The questionnaire we used to assess PE attitude may have contributed to these differences with prior research: although the PEAS questionnaire (Orlić et al., 2017) generally assesses teacher behaviors and the curriculum content in a manner that is similar to how the Student Attitude Toward PE scale (Subramaniam and Silverman, 2007; Hu et al., 2014; Silverman, 2017; Pereira et al., 2020) does, the latter focuses on the cognitive and affective components of PE attitude, whereas the PEAS also delves into the motivational/behavioral aspects of PE attitude. Thus, the increase in positive attitude toward PE that we found may have been explained by this comprehensive consideration of the four dimensions of PE attitude, which incorporate sources of information derived from students' feelings, beliefs, motivations, previous behaviors, and experiences stored in students' memory about the subject.

Moreover, in the Philippines, the PE curriculum comprises fitness concepts, games, sports, rhythms, and dance strands, with various topics divided into four quarters throughout the academic year (Department of Education, 2016). Thus, it is possible that, during the time of data collection (in the third quarter of the academic year), students were able to access more extensive memories regarding their recent lessons, activities, and the behaviors of teachers who provided them with these classes. In the data collection period, grade 10 Filipino students were having lessons about lifestyle, weight management, and dance activities (e.g., hip hop and cheer dance); grade 9 students were studying community fitness and ballroom dances; and grade 8 students were studying PA training guidelines and team sports. Since grade 10 students had higher mean scores for PE attitude, we can infer that they may have enjoyed their specific activities more than did students in the other two grades we analyzed. This noteworthy finding suggests that students' PE attitude tend to increase upon having a curriculum that is satisfying, interesting, and enjoyable for them; based on prior research, we infer that these feelings may be evoked when PE activities are challenging, useful, less arduous, and interactive, and the teacher is encouraging and friendly toward students. However, further studies are warranted to better understand why such a trend occurred.

The results of the analyses by student sex and PA outside school showed no statistically significant differences in the mean scores for general PE attitude and dimensions, thereby refuting hypotheses 3 and 4. The findings confirm some in previous studies (Subramaniam and Silverman, 2007; Scrabis-Fletcher et al., 2016; Marttinen et al., 2018), which found no sex differences in PE attitudes. However, the current results also contradict other studies, which revealed that female students (Zeng et al., 2011; Pereira et al., 2020) or male students (Koca and Demirhan, 2004; Koca et al., 2005; Mercier et al., 2017; Orlić et al., 2017) had more positive attitudes toward PE compared with their counterparts. Furthermore, our findings did not support the findings of studies that reported that students with PA outside school had more positive attitudes toward PE than those who did not (Orlić et al., 2017, 2018; Pereira et al., 2020).

The lack of significant differences for PE attitude by student sex and PA outside school may be attributed to students having experienced numerous PE lessons that were perceived as fun, meaningful, and engaging, and these interesting activities may even have been experienced outside school. Based on students' reports for this study, their PA outside school were mostly badminton, volleyball, running/jogging, and dancing (i.e., street dance and Zumba), which are all part of the PE curriculum in the Philippines and were likely to have been experienced in their PE classes. Summarizing, our results indicate that student sex and PA outside school may not be significant determinants of PE attitude and that both female and male Filipino students, regardless of PA activities outside school, viewed PE in a favorable way and at similar degrees.

Generally, while attitude scores for male teachers were more positive compared with those for female teacher, there were no significant differences in students' PE attitude by teacher sex, refuting our hypothesis 6. However, we did find a significant interaction between student sex and teacher sex: when their teacher was male, female students had a more favorable attitude toward PE than when the teacher was female; when the teacher was female, male students had a higher PE attitude than female students. Moreover, when the teacher was female, male students' satisfaction with PE was higher than that of female students, while when the teacher was male, female students' comfort in PE was higher than that of male students.

The significant findings may be explained by the gendering processes in PE (Berg and Lahelma, 2010; Lima et al., 2020) such as gender order (Connell, 1987), which creates power relation patterns between men and women in society and gender system (Hirdman, 1990) that categorizes males and females according to their differences in all aspects of life and follows a hierarchy based on male norms. Perhaps due to the physiological differences between male and female, wherein the former tends to be stronger, male PE teachers were likely to place lower expectations and demands on performances of female students and thereby provided more verbal interactions (Davis and Nicaise, 2011) and adjusted male-preferred activities to accommodate females. Accordingly, these behaviors of male PE teachers were perceived by female students to be helpful and encouraging that led to their increased positive attitude and comfort in PE. Conversely, following the male standard in PE, that is, students are expected to achieve certain performance levels comparable or exceeding the male model. In this case, female PE teachers could have provided more feedback and interaction to male students than females and led to the latter's lower satisfaction and positive attitude toward PE. This notion confirms a previous study (Nicaise et al., 2007) that showed female students perceived receiving less positive feedback compared with male students from female PE teachers despite their efforts and good performance. Nonetheless, these findings, therefore, highlight that student and teacher sex are factors that together, rather than independently, influence PE attitude. However, further explorations are warranted as to why this interaction pattern occurred.

To strengthen students' positive attitudes toward PE, PE teachers should consider how they interact with students of the same and different sex. Particularly, and based on the significant findings for the satisfaction and comfort dimensions of PE, female PE teachers should strive not to decrease the satisfaction level of female students during PE by striving to make the class fun and interesting and creating an atmosphere that facilitates social interactions, harmonious teacher–student relationships via positive feedback and technical information, and skill mastery. On the other hand, male teachers, when dealing with male students, should focus on making the class more comfortable through the proposal of less tiring and competition-focused activities and avoiding verbal and non-verbal criticisms. For school administrators, investing in new and modern equipment that are interesting for students and securing spaces both in and outside the vicinity of the school are noteworthy projects. Furthermore, providing a separate changing/dressing area where students can comfortably use is suggested since 58% and 60% of male and female students, respectively, reported being too uncomfortable to change clothes in front of others. Implementing these recommendations may support the formation of students' positive attitudes toward PE and promote more student engagement.

### Relationships Between PE Attitude and PE Dimensions

Results of the hierarchical regression showed that all dimensions of PE (i.e., satisfaction, comfort, activity, and teacher) were significant predictors of PE attitude in male students; thus, when PE lessons are motivating, satisfying, and stress-free, and when PE teachers display supportive and friendly behaviors, male students' positive attitude toward PE tends to increase. In female students, all PE dimensions and PA outside school were significant contributors to PE attitude; however, the contribution of PA outside school was negligible. Thus, female students tend to develop a negative attitude toward PE when they have no PA outside school, teachers who lack the skills to teach PE and display unsupportive behaviors, and/or a PE curriculum that is too stressful and too focused on competition.

These remarks underscore the importance of these variables for the development of both positive and negative attitudes toward PE for students of both sexes, confirming hypothesis 2; these results also concur with other reports on how curriculum and teacher behavior can impact students' PE attitude (Luke and Sinclair, 1991; Silverman, 2017). However, more explorations are needed to identify the factors influencing PE attitude outside these dimensions, such as students' psychological status, facilities, PE equipment, and specific behaviors of teachers (e.g., feedback and attention), all of which have been shown to relate to PE attitude (Luke and Sinclair, 1991; Nicaise et al., 2007; Silverman, 2017) but were not covered in this study.

Summarizing, our results showed that demographic factors accounted for <5% of the variance in predicting PE attitude, while the remaining 95% was significantly and uniquely explained by factors related to PE dimensions, which mostly comprised various affective, cognitive, and motivational components of the PE program and teacher behaviors.

## Limitations

The collected data were provided by students from one public school, in one city, in a metropolitan area in the Philippines; this implies difficulties regarding the generalization of our results to other areas (vs. rural) or school types (vs. private), suggesting the need for inclusion of these variables (school location and type) in future studies. Such future examinations will not only provide knowledge on students' attitude toward PE in rural and private schools but also highlight the influence of these factors on PE attitude. The large sample size and implementation of a standard curriculum content across major public schools in the city highlight the value of these findings.

Furthermore, our participants were only middle school students, meaning that the results are exclusive to this population. Future researchers should replicate this study with the inclusion of other grade levels (e.g., grades 11 and 12), as this will allow for examinations regarding the longitudinal trend of PE attitude (i.e., if the positive trend we observed would perpetuate). We would like to highlight the importance of further studying the topic in the Philippines.

## Strengths

Albeit we included influencing factors of PE attitude that have been commonly found in past literature (i.e., sex, grade level, PA outside school/sport participation), our study expanded prior research by the addition of teacher sex as a potential influencing factor. Previous studies were not able to examine this aspect (Zeng et al., 2011; Hu et al., 2014; Orlić et al., 2017; Pereira et al., 2020). Accordingly, we found that teacher sex moderated the relationship between student sex and PE attitude, providing a novel insight for the literature on the topic. However, more research is needed to examine whether the influence of teacher sex on PE attitude is direct or indirect, especially while considering teacher gender identity. We also examined various correlates of PE attitude by looking at their relative contribution in predicting PE attitude, something that was not analyzed in previous studies (Subramaniam and Silverman, 2007; Zeng et al., 2011; Hu et al., 2014; Orlić et al., 2017; Pereira et al., 2020).

Based on the MODE model, attitude refers to the overall association between the beliefs toward a target object and the degree of evaluations related to a target attitude object that are stored in memory, and these guide judgment and behavior when the strong attitude object is spontaneously accessed from one's memory. Hence, Filipino students' attitude toward PE may have come from their general and specific emotional experiences about and motivations to participate in PE, and their personal views about the teacher. By analyzing these sources of information, we found that Filipino students had a positive and relatively high attitude toward PE. Accordingly, if these strong and positive attitudes toward PE are accessed from memory, students may feel more likely to participate in PE and PA in and outside school when presented with this kind of immediate situation. The opposite is likely to occur for students with negative attitude toward PE; therefore, to avoid this undesirable response, students with such negative attitudes should be further motivated and given opportunity to engage in PE.

## Implications

To encourage students' continuous and active involvement in PE, particularly for those with strong and favorable attitudes, PE teachers are suggested to strive to have the following behaviors: be creative in their pedagogical strategies, resourceful and enthusiastic when introducing new sports and PAs, and passionate about the values and benefits of PE and PAs to their students. Such behaviors may allow for students to experience pleasant situations during PE, subsequently allowing for them to more easily and spontaneously recall such pleasantness related to PE.

Furthermore, for students who tend to instinctively avoid PE and PA, possibly owing to intense and unfavorable attitudes toward them, we recommend PE teachers to strive to be as considerate as possible when interacting with their students. For male students, teachers should focus on providing satisfying general emotional experiences (i.e., satisfaction dimension), giving them the opportunity to choose the PE activities that are interesting and enjoyable for them and ample time to play with their peers. For female students, teachers should consider improving their interactions with and overall presence by displaying proper behaviors related to health and fitness and providing ample feedback and encouragement when teaching. This is especially important for female students because the teacher dimension for this group yielded the lowest score among all dimensions of PE (mean = 1.50).

Further, students with a strong negative attitude toward PE should receive more efforts related to motivation to engage in PE and opportunities to deliberately experience and process new positive experiences; these may eventually turn into beliefs and change students' perceptions about PE for the better—consequently leading them to attempt to partake in PE once more. Finally, we recommend for school administrators to provide PE teachers with special training courses to enhance both their teaching and communication capabilities; doing so may allow for them to create a better PE curriculum and better connect with their students, thereby supporting the development of better attitudes toward PE in students.

## Conclusions

We found that students' PE attitude did not decrease as they got older, regardless of student sex. To our knowledge, this is the first study with this kind of result, providing a new perspective toward understanding PE attitude. Moreover, the study provides new insights into the role of teacher sex as a moderator in the relationship between student sex and PE attitude, and highlights the importance of PE curriculum—particularly the affective components of attitude—in predicting students' PE attitude.

Overall, Filipino middle high school students had strong positive views about their PE curriculum and their PE teachers, deeming them as valuable, enjoyable, and motivating. Teachers and school administrators should, therefore, create curricula and policies that do not let these positive attitudes decline; based on prior evidence, we can infer that the maintenance of these strong positive attitudes toward PE can lead students to healthy PA behaviors in the near future.

## Data Availability Statement

The raw data supporting the conclusions of this article will be made available by the authors, without undue reservation.

## Ethics Statement

Ethical review and approval was not required for the study on human participants in accordance with the local legislation and institutional requirements. Written informed consent to participate in this study was provided by the participants' legal guardian/next of kin.

## Author Contributions

AC, MK, and H-DK developed and designed the research project. AC collected the data and wrote the manuscript. H-DK and MK analyzed the data and made revisions in the manuscript. All authors contributed to the article and approved the submitted version.

## Conflict of Interest

The authors declare that the research was conducted in the absence of any commercial or financial relationships that could be construed as a potential conflict of interest.
